# Dry Mopping vs. Saline Irrigation of Gallbladder Fossa After Bile Spillage During Laparoscopic Cholecystectomy: Randomized Control Trial

**DOI:** 10.7759/cureus.13059

**Published:** 2021-02-01

**Authors:** Dujanah S Bhatti, Raheel Ahmad

**Affiliations:** 1 Plastic and Reconstructive Surgery, Aberdeen Royal Infirmary, Aberdeen, GBR; 2 Surgery, Holy Family Hospital, Rawalpindi, PAK

**Keywords:** area of interest lap gi surgery, gallstone disease (gsd)

## Abstract

Introduction

The laparoscopic approach, as compared to open cholecystectomy, is still considered the gold standard, despite a higher incidence of micro insults. The most common approach to treat spilled biliary contents and lost stones in laparoscopic cholecystectomy is the retrieval of the stone through an open approach, or laparoscopically, ending with a peritoneal wash and aspiration.

Material and methods

We conducted a double-blinded randomized controlled trial. In the study group, patients with bile spillage during cholecystectomy underwent suction of all spilled bile and evacuation of all visible stones followed by dry mopping of the gallbladder fossa with gauze swab through an epigastric port. In the control group, after suction of all bile and visible stones, the gallbladder fossa was washed with 250 ml of saline, and fluid was aspirated through the epigastric port.

Results

Sixty patients were included (30 patients in each group), 71.6% were female and the rest were male. There was a statistically significant difference in pain scores between the two groups (p=0.001). The dry mopping group had lower pain scores as compared to the other group postoperatively. The incidence of the intraabdominal collection in both groups are statistically insignificant, however, port site infection and intraabdominal collection are higher in the control group (irrigation group).

Conclusion

Although there is not much literature on the best approach to biliary spillage in laparoscopic cholecystectomy. We believe that dry mopping had better postoperative patient outcome as compared to the saline wash.

## Introduction

One of the most frequent complications of laparoscopic cholecystectomy is the iatrogenic perforation of the gallbladder and spillage of its content into the abdominal cavity [1]. Although, the complications of the open cholecystectomy and laparoscopic techniques are somewhat similar [2]. The laparoscopic approach is still considered the gold standard, despite a higher incidence of micro insults.

Contrary to recent data, laparoscopic cholecystectomy (LC) has a lower morbidity and mortality rate as compared to the open technique [3-4]. The long-term sequelae of biliary spillage on LC range from mild inflammation to reactive fibrosis and abscess formation [5-6]. Thus, many advocate conversions to the open method when such a complication happens [7]. Recent data suggest that this can be done by the retrieval of stones and either mopping the gallbladder fossa with surgical gauze or by an interoperative wash to evaluate the spilled content. International data suggest that the complications can range from 2.3% and 7% [8]. This incidence doubles when stones are not removed [9].

The most common approach to treat spilled biliary content and lost stones in LC is the retrieval of the stones through an open approach, or laparoscopically, ending with a peritoneal wash and aspiration. Of these, the latter is the most agreed upon in our region [10]. Every attempt should be made to retrieve all spilled stones, not to displace them to hidden areas, and end with a though wash and adequate aspiration. This method is widely practiced by the majority of surgeons [11-12]. This serves the purpose of diluting any infected bile and may allow the stones to be washed up into the suction system. An alternative is to dry mop the spilled content after laparoscopic removal of spilled stones. This method has not been studied much and limited data are available on its suitability and postoperative complications, especially pain. This prospective randomized study aimed to investigate the effects of peritoneal wash and aspiration versus the dry mopping of spilled biliary content during LC on the early and late postoperative course of patients.

## Materials and methods

Study design, setting, and duration

We conducted a double-blinded randomized controlled trial from January 2019 to December 2019 in Surgical Unit-I of Holy Family Hospital, Rawalpindi, Pakistan. Sixty patients who had bile spillage during laparoscopic cholecystectomy for gallstones were included in the study.

Operational definition

Pain Visual Analog Scale

A horizontal scale marked from zero to 100 after eight hours of surgery was used as a measure of the visual analog scale (VAS) for pain.

Inclusion and exclusion criteria

All patients between 16 and 60 years of age and having gallstones were included in the study. Those with empyema, mucocele, gangrenous gallbladder, or gallbladder malignancy were not included in the study. Moreover, patients with diabetes mellitus on medication, patients with underlying malignancies, patients taking steroids, and immunocompromised patients were also excluded from the study. The pediatric age group was excluded.

Sampling technique

Non-probability consecutive sampling was done, and all patients satisfying the inclusion criteria were included in our study. In Group A (study group), patients with bile spillage during cholecystectomy underwent suction of all spilled bile and evacuation of all visible stones followed by dry mopping of the gallbladder fossa with a gauze swab through the epigastric port. In Group B (control group), after suction of all bile and visible stones, the gallbladder fossa was washed with 250 ml of saline, and fluid was aspirated through the epigastric port. The gallbladder in both groups was removed from the umbilical port. In both groups, a single, one-off dose of broad-spectrum intravenous (IV) antibiotic was given intraoperatively.

To minimize the bias in our study, the patients and investigators were blinded to the allocation of patients to the study group and to the types of intervention done. Surgery was standardized in each case by the same team of surgeons, and preoperative and postoperative analgesia was also standardized in all patients. The completion of skin closure was considered as time point zero. A VAS score was administered to assess the pain four hours after surgery. Ports (excluding umbilical) were assessed on the fifth day and wound infection was described as having any discharge or redness around the wound causing pain, fever, or raised white blood cell (WBC) count. Patients with persistent pain, fever, or raised WBCs underwent an ultrasound scan of the abdomen by a consultant radiologist to see the presence of intra-abdominal collection.

Data analysis

For statistical analyses, data were analyzed in the Statistical Package for Social Sciences (SPSS) v19 (IBM Corp., Armonk, NY).

Categorical variables like gender were depicted as frequencies and percentages. Continuous variables, such as age, VAS (0-100), were explained as mean and standard deviation. An independent sample t-test was used to compare the mean VAS of both study groups at a 5% level of significance. A p-value of < 0.05 was considered statistically significant. A post-stratification independent sample t-test was applied. A p-value of=0.05 was taken as statistically significant.

## Results

Our study included a total of 60 patients. Table 1 shows the mean age of patients. Most patients were female, i.e., 71.6%, and the remaining 28.3% were male. Table 2 shows the detailed distribution according to gender. The mean pain scores of patients in the two groups are shown in Table 3. There is a statistically significant difference in pain scores between the two groups (p=0.001). The same pain scores according to gender are stratified in Table 4, which shows that both males and females in the dry mopping group had lower pain scores as compared to the other group. Table 5 shows the incidence of wound infection and intra-abdominal collection in both groups, which is statistically insignificant; however, port site infection and intra-abdominal collection are a bit high in the control group (irrigation group).

**Table 1:** Comparison of age with study groups

		Study Groups	
		Group A	Group B
Age (years)	n	30	30
	Mean	39.62	39.06
	SD	12.73	13.25

**Table 2:** Comparison of gender with study groups

		Study Groups		Percentage	Total
		Group A	Group B		
Gender	Male	09	08	28.3%	17
	Female	21	22	71.6%	43
Total		30	30	100%	60

**Table 3:** Comparison of pain score with study groups

		Study Groups		Total
		Group A	Group B	
Pain score	n	30	30	60
	Mean	1.41	3.61	Ind. t test=-6.12 p-value=0.001*
	SD	1.26	1.94	

**Table 4:** Comparison of pain score with study groups stratified by gender

Pain score	Gender	Study Groups		p-value
		Group A	Group B	
	Male	1.62±1.27	3.54±1.94	0.001*
	Female	1.58±1.28	3.67±1.97	0.001*

**Table 5:** Comparison of intra-abdominal collection and port site infection with study groups

		Study Groups		Total	
		Group A N=30	Group B N=30		
Abdominal collection	Yes	01	02	03	p-value=0.071
	No	29	28	57	
Port site infection	Yes	00	02	02	p-value=0.069
	No	30	26	58	

## Discussion

This randomized controlled trial elucidates the comparison of patient outcomes of two techniques used to address biliary spillage during laparoscopic cholecystectomy, along with the postoperative complications that develop because of biliary spillage. Furthermore, there is a two-fold increase in the complications associated with biliary spillage post-laparoscopic cholecystectomy, and it is often missed by most surgeons [13]. Unlimited data is available on the comparison of dry mopping versus saline irrigation post biliary spillage in LC [14-15]. We took a group of patients and applied this technique through a randomized control trial and saw a statistically significant difference in pain score postoperatively. However, the long-term complications of gallbladder perforation and spillage of its content into the peritoneal cavity are still under investigation [16]. Previous studies showed that the incidence of perforation during LC ranges from 8 to 39.9 (mean 20). The successful retrieval of gallstones is only possible in approximately 63% of the cases [4].

Postoperative VAS

Judging from our experience, there was a statistical difference in the postoperative VAS among the two groups of patients. In Group A, in which we employed the technique of stone retrieval followed by dry mopping with a surgical gauze, the VAS score was mean: 1.41 (SD: 1.216) compared to Group B in which irrigation was employed after BS where the VAS was 3.61 mean (SD: 1.94). This statistical significance (p=0.0001) shows that dry mopping has better postoperative pain outcomes as compared to saline irrigation, which is the treatment of choice among the majority of the surgeons in LC. Ideally, there would be a disparity if such patients were instituted and, indeed, this would have had a detrimental effect because of the influence of various other factors on the postoperative outcome. However, our surgeries were uniformed by the patient’s history, clinical presentation, same operative surgical team, and they unanimously agreed upon postoperative treatment [17]. Various studies advocate that postoperative pain is a consequence of the complications associated with gallbladder perforation and the spillage of its contents such as abdominal wall abscess, bronchiectasis, lung abscess, subdiaphragmatic abscess, liver abscess, splenic abscess, and so on [17]. However, the time duration of such complications to develop a significant effect on the patient’s morbidity could range from a few months to up to 20 years [18]. Although studies advocate that LC pain levels are significantly lower when compared to conventional open technique and the required analgesia is considerably reduced in LC (mean VAS score 3.8 vs 7.7) [19].

Postoperative pain after LC starts from the first day with its peak and gradually subsides after two to three days [20], and they reported 80% of patients asking for analgesia postoperatively [21]. The use of sodium bicarbonate for a peritoneal wash has shown significant improvement in postoperative VAS. Recent researches have shown that peritoneal acidosis caused by CO_2_ insufflation is the main cause of peritoneal acidosis, therefore, sodium bicarbonate washout may neutralize the effect of acid milieu on the peritoneal cavity and, therefore, reduced postoperative pain [21]. We utilized normal saline irrigation and suction for the spillage, however, the possibility of employing sodium bicarbonate peritoneal wash after spilled biliary content may contribute to a further reduction in postoperative pain.

Postoperative intraperitoneal collection and abscess

Statistically and from previous studies, there is no correlation between fluid collection post LC or by the conventional method [22-23]. Limited data are available on the outcome of abdominal collection in a port site infection after dry mopping versus saline irrigation post biliary spillage. In our study, only one patient from Group A experienced abdominal collection postoperatively, whereas in Group B, two patients had abdominal collection and two patients had a port site infection following saline irrigation. Although these results are statistically insignificant, we must appreciate the low incidence of complications following dry mopping after stone retrieval post biliary spillage.

## Conclusions

Postoperative pain score and complications carry a significant burden on surgical units that practice laparoscopic cholecystectomy. This can be significantly reduced by adopting practices that render the patient comfortable and in a better state. Although there is not much literature on the best approach to biliary spillage in laparoscopic cholecystectomy. we believe that dry mopping had better postoperative patient outcomes as compared to saline wash.
